# Potential role of M2 TAMs around lymphatic vessels during lymphatic invasion in papillary thyroid carcinoma

**DOI:** 10.1038/s41598-020-80694-3

**Published:** 2021-01-13

**Authors:** Takanobu Kabasawa, Rintaro Ohe, Naing Ye Aung, Yuka Urano, Takumi Kitaoka, Nobuyuki Tamazawa, Aya Utsunomiya, Mitsunori Yamakawa

**Affiliations:** Department of Diagnostic Pathology, Faculty of Medicine, Yamagata University, 2-2-2 Iida-Nishi, Yamagata, 990-9585 Japan

**Keywords:** Tumour immunology, Cancer microenvironment

## Abstract

The aim of this study was to examine whether lymphatic invasion in papillary thyroid carcinoma (PTC) occurs when tumour-associated macrophages (TAMs) injure lymphatic vessels together with cancer cells. While there was no difference in the lymphatic vessel density in PTC and follicular thyroid carcinoma (FTC), the number of TAMs around the lymphatic vessels was increased in PTC compared to that in FTC. In particular, TAMs were observed together with cancer cells in lymphatic invasive lesions, and the number of M2 cells inside and outside the lymphatic vessels showed a significant correlation. MMP-2 mRNA was expressed in nonneoplastic stromal cells as well as cancer cells, and double immunofluorescence staining confirmed M2 positivity. Consequently, this study reveals that M2 TAMs around lymphatic vessels within the tumour border of PTC may be associated with the lymphatic invasion of cancer cells. This study represents a step forward in elucidating the mechanism of lymphatic invasion.

## Introduction

Papillary thyroid carcinoma (PTC) and follicular thyroid carcinoma (FTC) are well-differentiated malignancies derived from the thyroid follicular epithelium^1^. In general, PTC metastasizes lymphatically to the cervical lymph nodes, while FTC metastasizes haematogenously to distant sites, such as bone and soft tissue, often several years after excision of the primary lesion. The frequency of lymph node metastasis is 17.3–78.0% for PTC^2^ and 1.9% for FTC^3^. The mechanisms of these opposing modes of metastasis have not been fully elucidated.

The lymphatic metastasis of cancer cells requires step-by-step lymphatic vessel invasion at the primary site. That is, cancer cells undergo epithelial-mesenchymal transition, a reduction in cell-to-cell connectivity, destruction of the basement membrane under the epithelium, and finally infiltration into pre-existing and newly formed lymphatic vessels produced by the tumour^4^. In addition to cancer cells, the cancer microenvironment has heterogeneous cellular and stromal components, such as macrophages, immunocompetent cells (e.g., lymphocytes and dendritic cells), fibroblasts, vascular endothelial cells, and the extracellular matrix. Macrophages are classified as M1 and M2 according to their functions^5^. M1 cells promote inflammation and immunity, protect against bacterial infection, prevent tissue injury, and suppress tumour growth, while M2 cells suppress inflammation and anticancer immunity and play important roles in angiogenesis and tumour progression. These facts show that M1 and M2 macrophages play bipolar roles in inflammation and immunity. Markers of M1 cells include inducible nitric oxide synthase, HLA-DR, CD80, CD86, CD169, and Toll-like receptors 2 and 4, and those of M2 cells include CD163, CD204, CD206, and arginase 1^5^. In addition, the transcription factors pStat1 and c-Maf are upregulated in M1 and M2 cells, respectively^6^. Macrophages existing in cancer tissue are referred to as tumour-associated macrophages (TAMs); in particular, TAMs with M2 characteristics (M2 TAMs) promote cancer cell proliferation and angiogenesis and suppress anticancer immunity. A variety of tumours with a high density of M2 TAMs have a poor prognosis, and thyroid cancer also shows TAM involvement in cancer immunity^7,8^. To the best of our knowledge, only two previous studies have demonstrated a relationship between TAMs and lymphatic invasion in thyroid cancer. That is, CXCL-8 secreted by macrophages makes it easy for cancer cells to invade lymphatic vessels^9^, and PTCs expressing decoy receptor-3 promote differentiation of TAMs into M2 cells^10^. Furthermore, M2 TAMs secrete matrix metallopeptidase (MMP) to destroy the extracellular matrix, and cancer cells can infiltrate the resulting void^11^. MMPs are enzymes that damage the extracellular matrix, which includes various types of collagen, and 23 types of MMPs have been reported in humans^12^. In particular, MMP-2 and MMP-9 play important roles in cancer infiltration^13^. However, it is unclear whether TAMs are involved in the lymphatic invasion of thyroid cancer cells.

We hypothesized that "lymphatic invasion in PTC occurs because TAMs injure the lymphatic vessels together with cancer cells". This study revealed for the first time in tissue sections that M2 TAMs around the lymphatic vessels within the tumour border of PTC may be associated with the lymphatic invasion of cancer cells.

## Results

### Comparison of the number of lymphatic vessels in PTC and FTC

**Figure 1:**
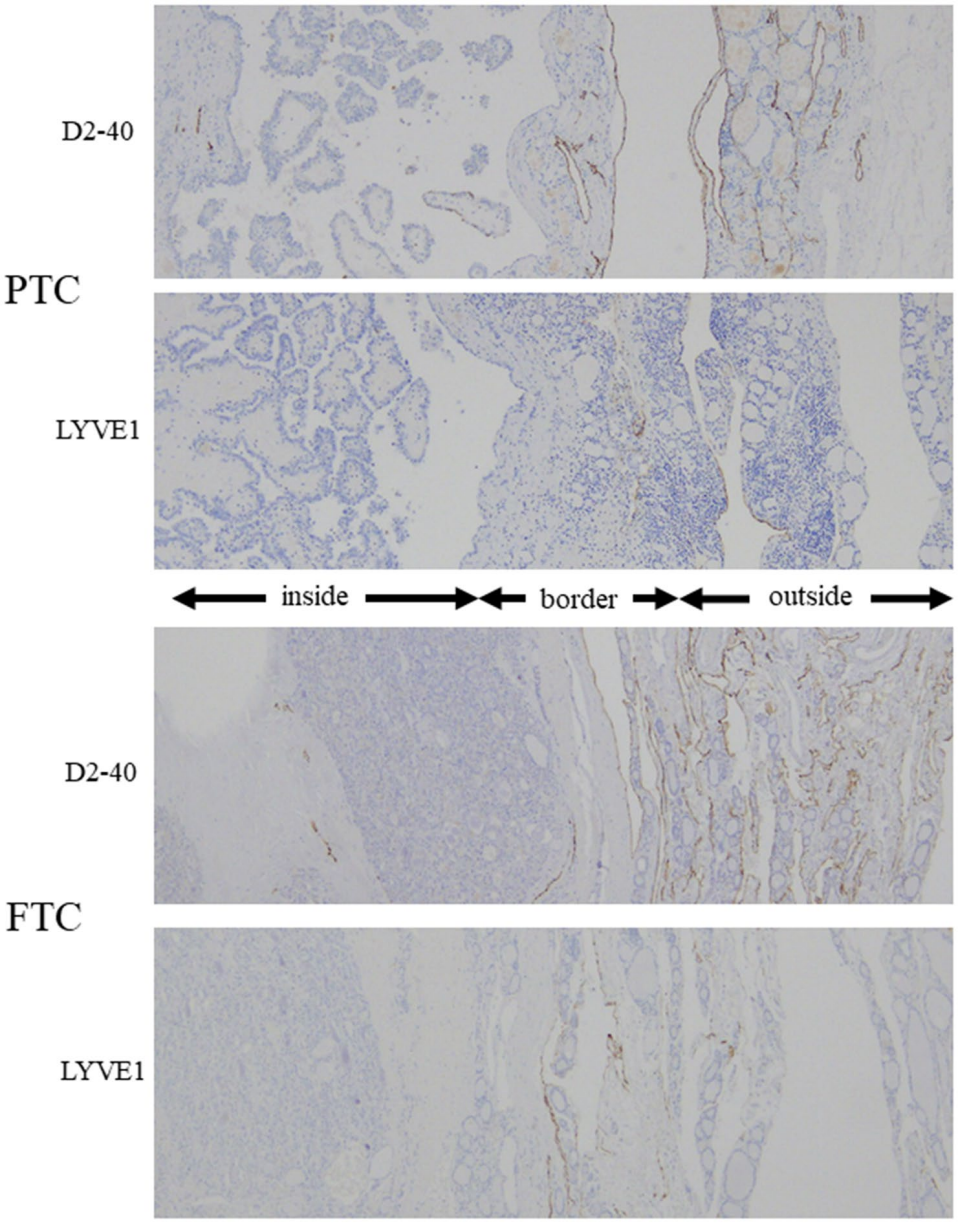
The distribution of D2-40^+^ and LYVE1^+^ cells in PTC and FTC. D2-40^+^  and LYVE1^+^ lymphatic vessels are often found in the border area or the outside area. *inside*; inside area, *border*; border area, *outside*; outside area.

**Figure 2:**
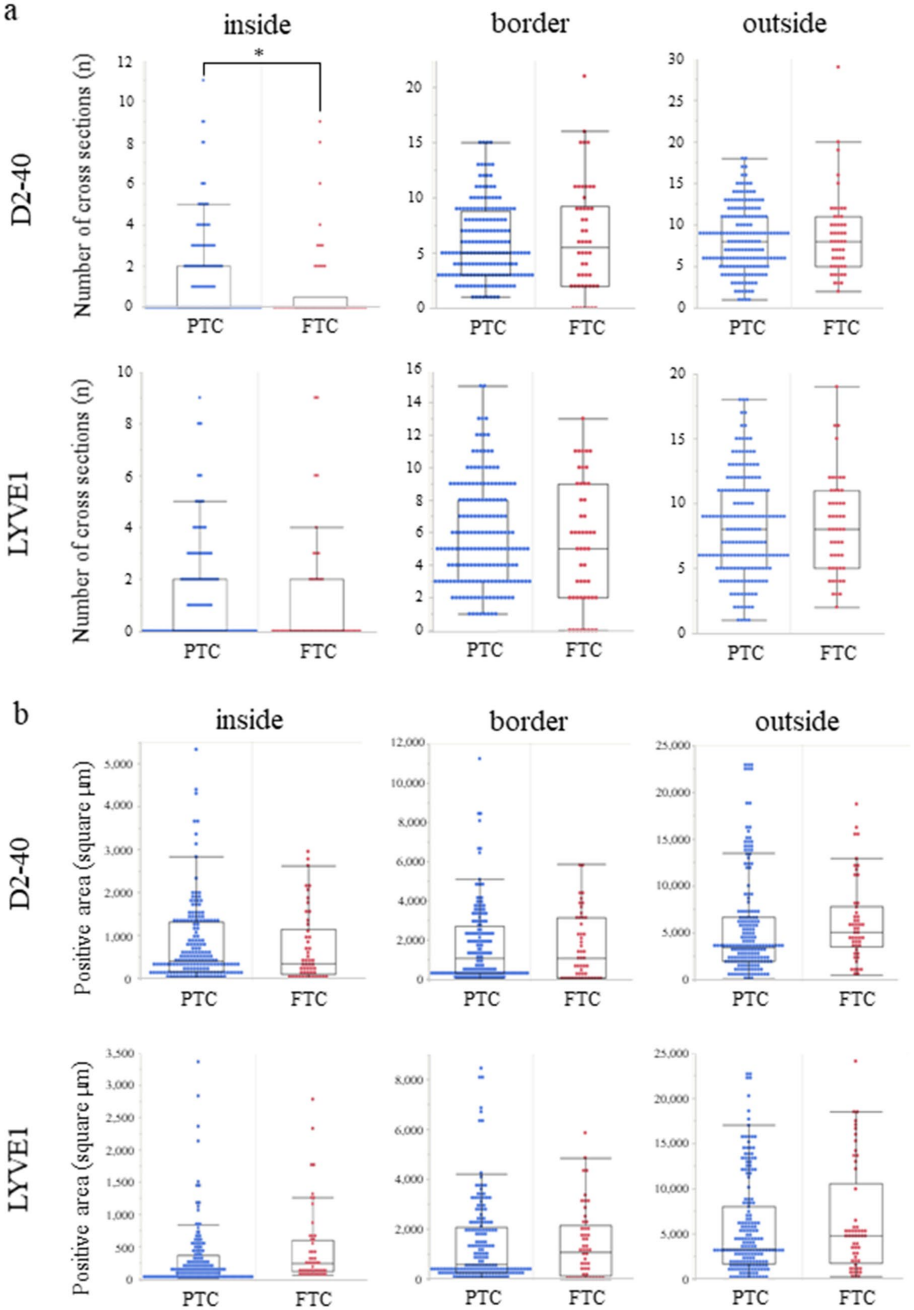
Comparison of lymphatic vessels in PTC and FTC. The number of cross-sections of D2-40^+^ and LYVE1^+^ lymphatic vessels was counted (**a**). Only the number of D2-40^+^ lymphatic vessels in the inside area was increased in PTC compared to FTC. Calculation of D2-40^+^ or LYVE1^+^ lymphatic endothelial areas using Halo analysis showed no difference between PTC and FTC at any location (**b**). *inside*; inside area, *border*; border area, *outside*; outside area.

We performed this examination to show that the cause of PTC susceptibility to lymph node metastasis is independent of lymphangiogenesis. The distribution of D2-40^+^ and LYVE1^+^ lymphatic vessels, as detected by single immunohistochemistry (IHC), in PTC and FTC is shown in Fig. 1. The number of lymphatic cross-sections is compared in Fig. 2. The *p* values were as follows: D2-40^+^ number of cross sections; inside area (*p* = 0.019), border area (*p* = 0.787), outside area (*p* = 0.938); LYVE1^+^ number of cross sections; inside area (*p* = 0.081), border area (*p* = 0.273), outside area (*p* = 0.930). Only the number of D2-40^+^ lymphatic vessels in the inside area was increased in PTC compared to that in FTC (Fig. 2a). Analysis of the IHC-positive area using HALO imaging analysis software (Indica Labs, Corrales, NM) did not show any significant differences. The *p* values were as follows: D2-40^+^ area; inside area (*p* = 0.112), border area (*p* = 0.983), outside area (*p* = 0.622); LYVE1^+^ area; inside area (*p* = 0.070), border area (*p* = 0.768), outside area (*p* = 0.424). (Fig. 2b).

### Comparison of the number of TAMs around the tumour border lymphatic vessels in PTC and FTC

**Figure 3:**
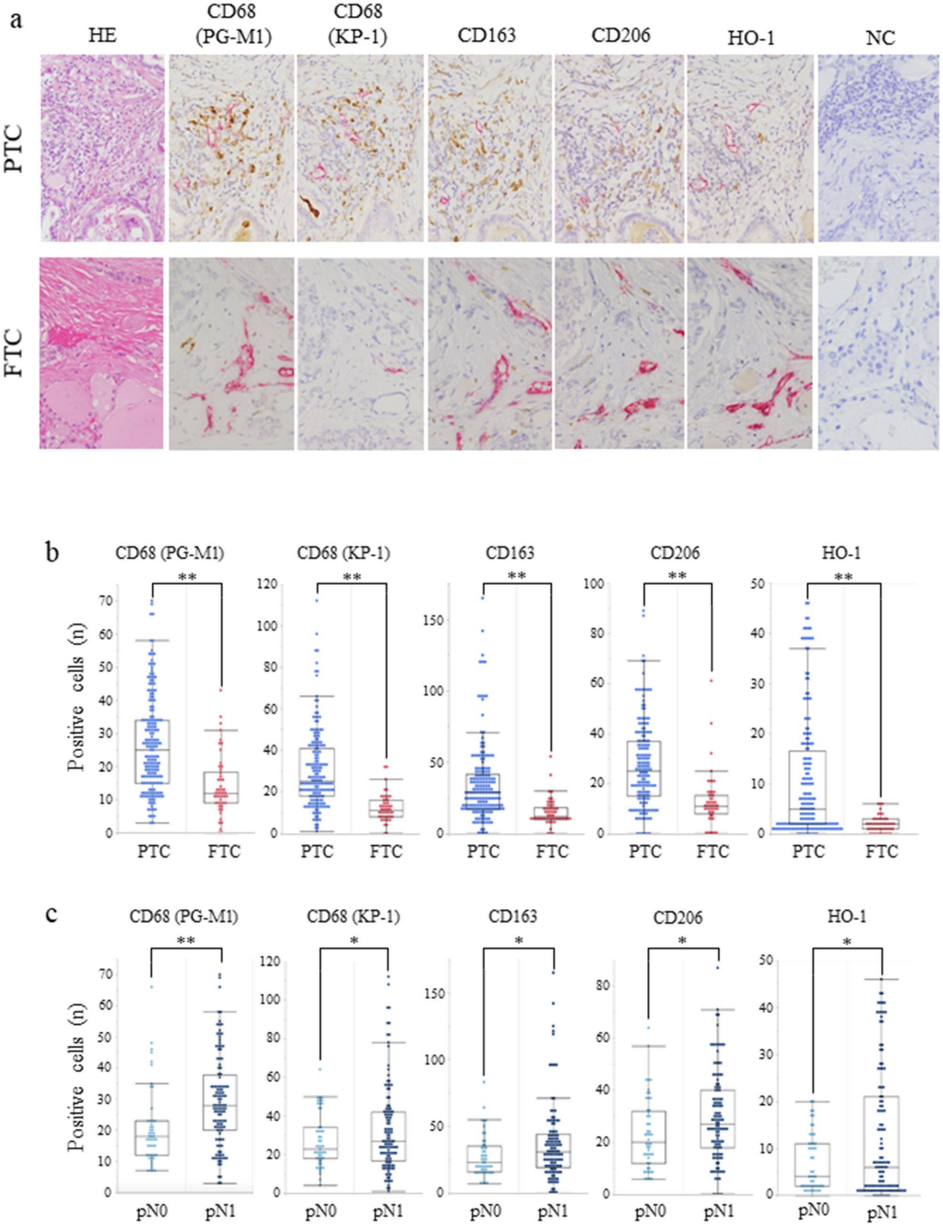
Macrophages around lymphatic vessels identified by D2-40 immunostaining in the border area of PTC and FTC. Macrophages positive for CD68 (PG-M1 and KP-1), CD163, CD206 and HO-1 are shown in *brown,* and lymphatic vessels positive for D2-40 are shown in *red* (counterstained with haematoxylin) (**a**). The number of macrophages positive for any marker was increased in PTC compared to FTC (**b**). Comparing the groups with and without lymph node metastases (pN0 and pN1) in PTC, there was significantly more perilymphatic macrophage infiltration in the group with lymph node metastases (**c**). NC, negative control, *p < 0.05, **p < 0.01.

A comparison of the macrophage counts around the lymphatic vessels in PTC and FTC is shown in Fig. 3. Considering the importance of lymphatic invasion at the tumour margin for lymph node metastasis, we noted that inflammatory cell infiltration in the tumour margin is more common in PTC than in FTC. In addition, the lymphatic vessels need to be destroyed for lymphatic invasion to occur, and macrophages are thought to play a role in this mechanism. The number of positive cells for each macrophage marker in PTC was as follows: CD68 (PG-M1, 26.90 ± 14.57), CD68 (KP-1, 30.31 ± 19.00), CD163 (30.07 ± 12.46), CD206 (27.24 ± 17.06), and HO-1 (10.57 ± 12.29). On the other hand, the number of positive cells for each macrophage marker in FTC was as follows: CD68 (PG-M1, 14.12 ± 9.18), CD68 (KP-1, 12.12 ± 6.49), CD163 (14.74 ± 9.36), CD206 (12.88 ± 10.44), and HO-1 (1.92 ± 1.61) (Fig. 3a). The number of positive cells for any macrophage marker in PTC was higher than that in FTC (*p* < 0.01, Fig. 3b). In addition, when comparing the groups with and without lymph node metastasis in PTC, there was significantly more macrophage infiltration around the tumour border in the group with lymph node metastasis [PG-M1 (*p* = 0.004), KP-1 (*p* = 0.035), CD163 (*p* = 0.032), CD206 (*p* = 0.016), and HO-1 (*p* = 0.013)] (Fig. 3c).

### Correlation of the number of TAMs inside and outside the areas of lymphatic invasion in PTC

**Figure 4:**
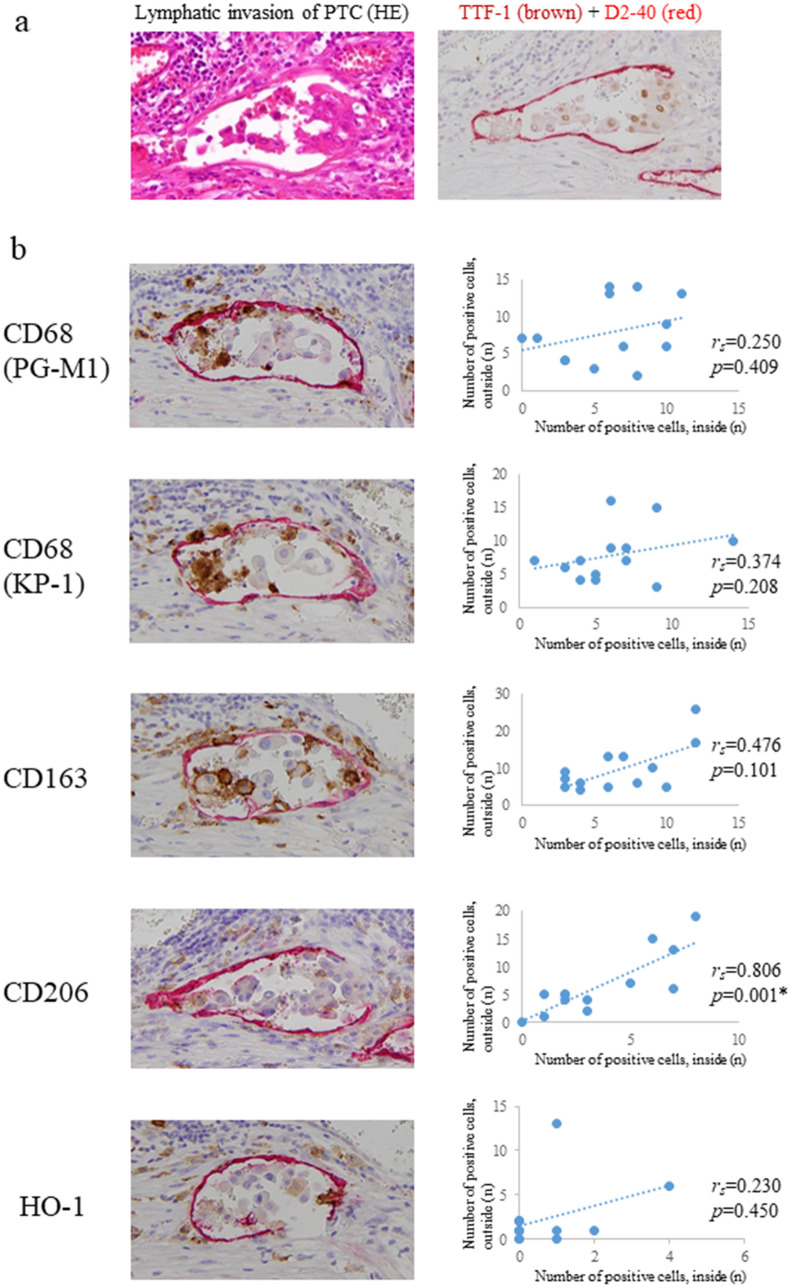
Lymphatic invasion (D2-40, *red*) and surrounding macrophages in PTC. Lymphatic invasion was identified by positive images of TTF-1 (**a**). PG-M1, KP-1, CD163, CD206, and HO-1 (*brown*) at the same site of lymphatic invasion in PTC. Comparing the number of positive macrophage markers inside and outside of areas of lymphatic invasion, we found a positive correlation only for CD206. (**b**). (counterstained with haematoxylin, **p < 0.01).

There are two possible origins of macrophages present during the lymphatic invasion of cancer. That is, they may have originally been present in the lymphatic vessels, or they may have invaded with the cancer cells from outside the lymphatic vessels. To show that TAMs in lymphatic invasion may have infiltrated from outside the lymphatic vessels, we examined the correlation of macrophage markers inside and outside the area of lymphatic invasion. The results are shown in Fig. 4. Lymphatic invasion in PTC was identified in 13 of 36 PTCs (Fig. 4a). Macrophages positive for any macrophage marker (PG-M1, KP-1, CD163, CD206, and HO-1) were found inside and outside the areas of lymphatic cancer invasion. The correlation of the number of positive macrophages inside and outside the areas of lymphatic invasion in PTC was as follows: PG-M1 (*rs* = 0.250, *p* = 0.409), KP-1 (*rs* = 0.374, *p* = 0.208), CD163 (*rs* = 0.476, *p* = 0.101), CD206 (*rs* = 0.806, *p* = 0.001), and HO-1 (*rs* = 0.230, *p* = 0.450) (Fig. 4b). Only the M2 marker CD206 was positively correlated inside and outside the area of lymphatic invasion.

### Detection of M2 and non-M2 cells inside and outside the areas of lymphatic invasion by triple IHC

**Figure 5:**
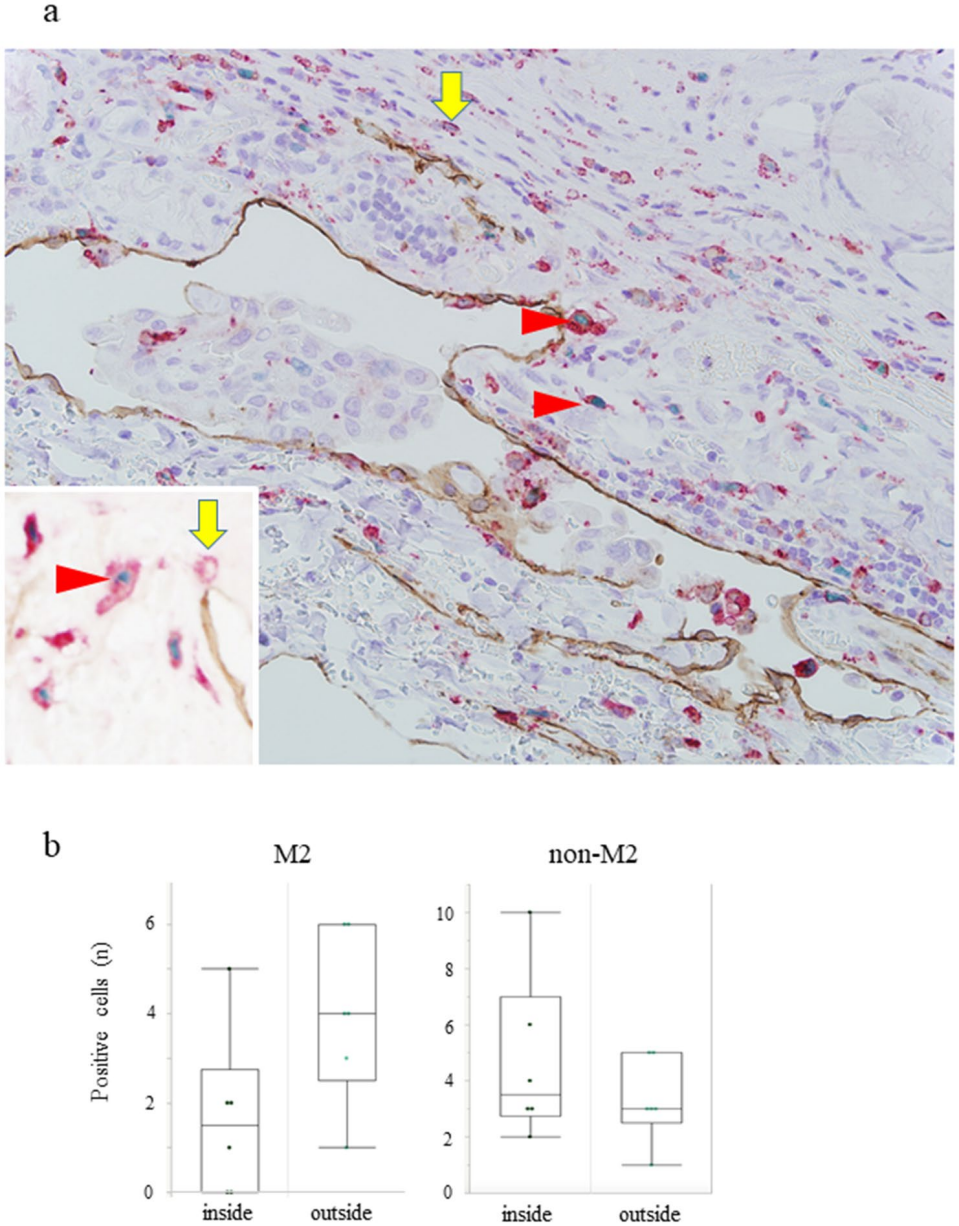
Triple IHC of the panmacrophage marker (*red*), D2-40 (*brown*), and c-Maf (nuclear stain, *green*) in PTC. A few panmacrophage markers (CD68 + CD163)^+^c-Maf^+^ M2 coexisted with cancer cells in D2-40^+^ lymphatic vessels (counterstained with haematoxylin) (**a**). The inset shows a typical M2 and non-M2 image without haematoxylin counterstaining. There was no difference in the distribution of M2 and non-M2 cells inside and outside the areas of lymphatic invasion (**b**). arrowhead; M2 cells, arrow; non-M2 cells.

We compared macrophages inside and outside the area of lymphatic invasion by the detection of an additional different M2 marker from the previous examination. The results are shown in Fig. 4. In 7 specimens in which lymphatic invasion was detected with haematoxylin and eosin staining, the invasion site disappeared after slicing the paraffin-embedding tissue and could not be examined. Finally, lymphatic invasion was detected in 6 specimens (Fig. 5a). There was no difference in the numbers of M2 and non-M2 cells either inside or outside the areas of lymphatic invasion (M2: *p* = 0.090, non-M2: *p* = 0.507, Fig. 5b). The number of eligible cases is small and may not be sufficient.

### Expression of MMP-2 mRNA according to RT-PCR and in situ RT-PCR and detection of MMP-2 protein in M2 cells by double IHC

**Figure 6:**
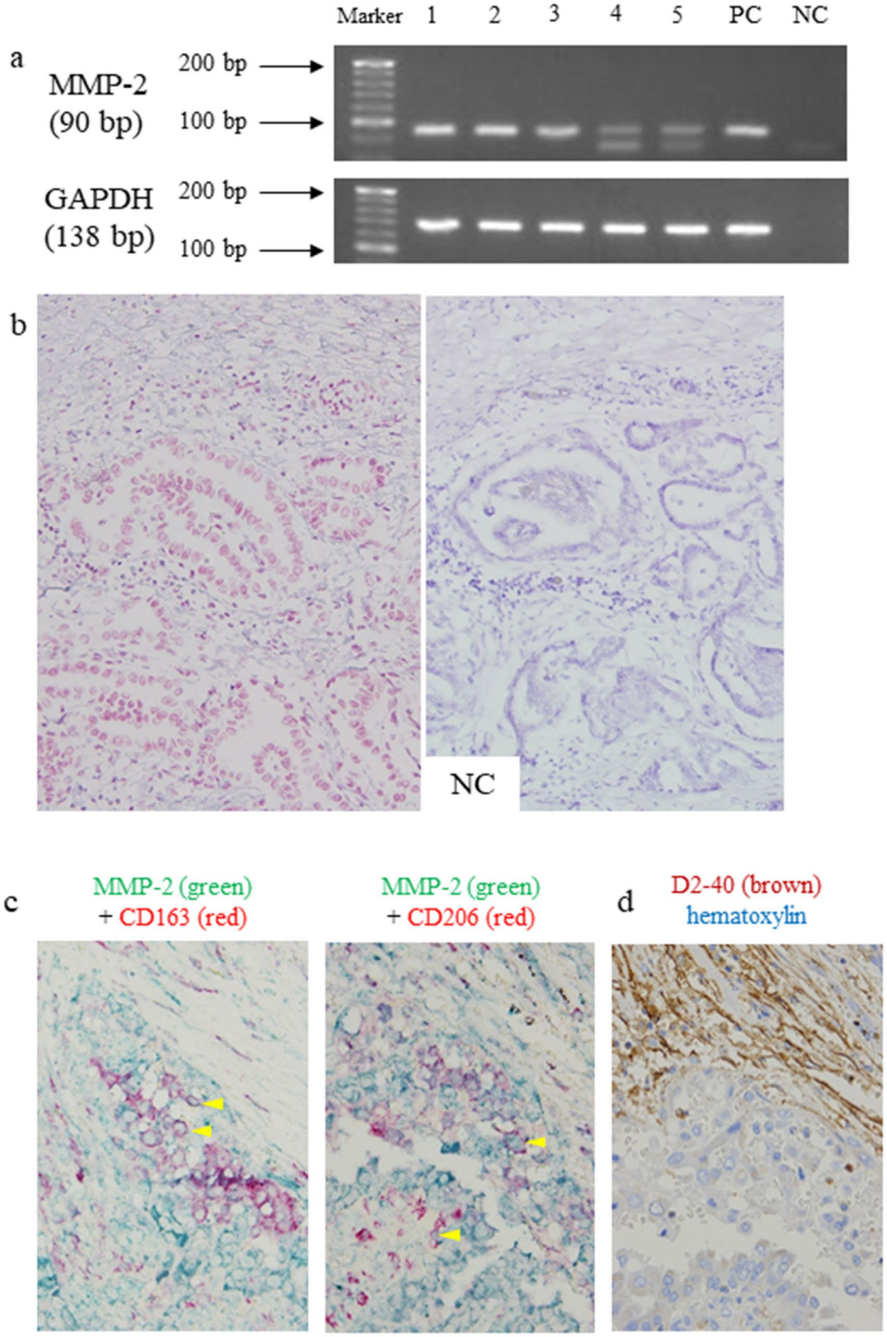
MMP-2 expression of M2 macrophage in tumour border. RT-PCR of MMP-2 and GAPDH in papillary thyroid carcinoma (PTC) (**a**). The expression of MMP-2 mRNA (90 bp) and GAPDH mRNA (138 bp) was observed in all 5 PTC patients by RT-PCR. Lanes 1–5: PTC patients. In the MMP-2 in situ RT-PCR, a positive red-coloured image was also obtained for the non-neoplastic stromal cells in the cancer border. An identical negative control is shown (**b**) (counterstained with haematoxylin). Double IHC of MMP-2 and M2 markers in the tumour border of PTC. Their coexpression and IHC of D2-40 in the same locations are shown. (No counterstaining in the double IHC) (**c**). *arrowhead*; M2 cells, NC; negative control, PC; positive control.

The examination of MMP-2 expression in macrophages is shown in Fig. 6. MMP-2 may be a protein that disrupts the basement membrane of lymphatic vessels. RT-PCR revealed a band corresponding to MMP-2 and GAPDH mRNA in all 5 PTCs (Fig. 6a). Based on the quality of the mRNA in the paraffin blocks, the five most recent cases among the eligible cases were selected. In situ RT-PCR performed on 3 of 5 specimens (cases 1, 2 and 3) revealed positive PTC cells and nonneoplastic cells in the border (Fig. 6b). MMP-2 protein expression in M2 TAMs is shown in Fig. 6c. We performed double IHC to detect MMP-2 and M2 markers in five cases where RT-PCR was performed. Double IHC staining revealed MMP-2 positivity not only in cancer cells involved in lymphatic invasion but also in CD163^+^ and CD206^+^ M2 TAMs in all 5 cases.

## Discussion

We initially examined the density of lymphatic vessels around the tumour border as a cause of frequent lymph node metastasis in PTC. There was no difference in the density of lymphatic vessels in two areas (the border area and the extratumoural area) between PTC and FTC. Only in the intratumoural area did the number of D2-40^+^ lymphatic vessels differ. A previous report demonstrated increased lymphatic vessel density in PTC rather than FTC, which is the follicular variant of PTC, and recurrent PTC^14^. Although some of the results of this study were similar, some of the results showed differences. We believe that the difference in the results of the study lies in the difference in the area where the lymphatic vessels were examined. That is, the other study examined intra- and extratumour tissues, whereas we examined the three regions. There was no difference in the number of lymphatic vessels within the tumour, at the tumour border, or outside the tumour between PTCs and FTCs, except for the number of D2-40^+^ cross sections. We cannot suggest that lymphangiogenesis does not contribute to the frequency of lymph node metastasis, but lymphatic invasion is often identified in the tumour margin on sections. In the present study, there was no difference in the tumour margin, suggesting that factors other than lymphangiogenesis may contribute to the frequency of lymph node metastasis.

In the next step, we focused on TAMs. According to a PubMed search, only one study on the diffuse sclerosing variant of PTC has examined the relationship between lymphatic invasion in PTC and TAMs^10^. A significant increase in TAMs was observed in the tumour border of PTC compared to that of FTC. The expression of the five macrophage markers examined (PG-M1, KP-1, CD163, CD206, and HO-1) in this study was increased in PTC, considering that both M1 and M2 cells were increased (Fig. 3). In one study, HO-1 was considered a marker of Mox cells (different from M2 cells)^15^, but in another study, M2 cells were reported to express HO-1^16^. It was also shown that the more macrophages there were around the lymphatic vessels, the more likely they developed lymph node metastasis. Although we could not show a difference between M1 and M2 macrophages, this may be one of the reasons why macrophages promote the lymphatic invasion of PTC. In addition, when comparing the positive and negative lymph node cases of PTC, there was an increase in the number of macrophages in the positive lymph node cases. This fact may be another piece of evidence for the contribution of TAMs to lymph node metastasis.

Furthermore, because we found a significant difference in the frequency of TAMs in the tumour border of PTC compared to FTC, for the first time, we focused on TAMs and their subtype inside and outside lymphatic vessels with cancer invasion in the border areas and the extratumoural areas in PTC. In lymphatic invasion in PTC, one or more intralymphatic macrophages often coexist with cancer cells (Fig. 4). Intralymphatic M2 cells are frequently observed in areas of lymphatic invasion when there are many perilymphatic TAMs, but the tendency has not been observed in the case of M1 cells. Furthermore, triple immunohistochemistry showed the presence of intralymphatic M2 TAMs in areas of lymphatic invasion, although the number of examined specimens was small and was not examined statistically. The two types of intralymphatic macrophages in areas of lymphatic invasion include macrophages already present in the lymphatic vessel and macrophages invading from the perilymphatic stroma in association with lymphatic cancer invasion. The latter may be because only M2 TAMs inside and outside lymphatic vessels were correlated with cancer invasion in this study. M2 TAMs may invade the lymphatic wall together with cancer cells, and they coexist with cancer cells in lymphatic vessels. One study reported that cancer cells infiltrated the collagen membrane when they were cocultured with macrophages in an experimental system using cultured human breast cancer cells, although the cancer cells alone did not infiltrate the collagen membrane^17^. The results of this study are similar, and it is possible that cancer cells infiltrate the basement membrane of lymphatic vessels by coexisting with macrophages. In addition, in Hodgkin lymphoma, the more CD206-positive TAMs are present, the more tumour tissue remodelling advances and then promotes dissemination^18^. Although the M1/M2 polarity of macrophages cannot be simplified, there are many other reports showing that M2 TAMs act as tumour promoters, and we also believe that M2 TAMs have a tumour-promoting effect in PTC.

In this study, the expression of MMP-2 mRNA and protein was found in TAMs together with cancer cells. MMP-2 is also called gelatinase A and is an enzyme that degrades type IV collagen, a component constituting the basement membrane. The expression of MMP-2 has been reported in macrophages in abdominal aortic aneurysms and in alveolar macrophages in pneumothorax^19,20^. On the other hand, Wu et al.^21^ demonstrated the increased expression of MMP-2 in PTC cells, and in other carcinomas, the cancer cells themselves secreted and damaged the extracellular matrix. Although there have been few reports of its expression in TAMs thus far, according to the results of this study, both cancer cells and TAMs express MMP-2 and damage the basement membrane of lymphatic vessels, such as macrophages found in inflammatory diseases.

The limitation of this study is that prognostic factors, such as extracapsular invasion and tumour diameter, were not considered. Although there is no specific opinion regarding PTC, the increase in TAMs generally has a poor prognosis in many patients^5,8^ and may be confounded with a prognostic factor, such as tumour size^22^.

Consequently, this study revealed for the first time that M2 TAMs around lymphatic vessels within the tumour border of PTC may be associated with lymphatic invasion. This study provides insights into the mechanism of lymphatic invasion, and it may be possible to identify drug targets that inhibit lymph node metastasis in the future.

## Methods

### Tissue specimens

We examined specimens from 36 patients with PTC diagnosed pathologically between 2010 and 2017 and 10 patients with FTC diagnosed pathologically between 2004 and 2017 at Yamagata University Hospital. We selected tissue sections that included the border between the cancer and the background thyroid gland for PTC and tissue sections exhibiting capsular or vascular invasion for FTC. The brief clinicopathological features are summarized in Table 1. Excised tissues were fixed in 10% buffered formalin for 12–48 h at room temperature and embedded in paraffin.

**Table 1:** The clinicopathological features of the patients with PTC and FTC.

	PTC	FTC
Age at diagnosis (years)	55.9 ± 13.9	63.2 ± 17.0
Sex, male, n (%)	13/36 (36.1)	4/10 (40.0)
Tumour size (cm)	1.92 ± 0.99	4.29 ± 1.28
Extrathyroidal extension, n (%)	20/36 (55.6)	1/10 (10.0)
Lymph node metastasis, n (%)	24/34 (70.6)	2/4 (50.0)
Distant metastasis, n (%)	1/36 (2.78)	5/10 (50.0)

### Ethical issues

This study was approved by the Ethical Review Committee of Yamagata University Faculty of Medicine (H29-303) and was performed in accordance with the Declaration of Helsinki. The Ethical Review Committee decided that this study did not need written informed consent because information regarding individual features from which others can identify the patient was excluded. Furthermore, we opted out in substitution for written informed consent. All the methods were carried out in accordance with the approved guidelines.

### Single IHC

Single IHC was performed as previously described^23^. Three-micrometre-thick sections were deparaffinized. Endogenous peroxidase activity was blocked with methanol containing 0.3% hydrogen peroxide for 30 min on ice. Antigen retrieval was performed using ethylenediaminetetraacetic acid (Antigen Retrieval Solution pH 9; Nichirei Biosciences, Tokyo, Japan) or citric acid (Antigen Retrieval Solution pH 6; Iatron Laboratories, Inc., Tokyo, Japan) in an autoclave (2 atmospheres, 121 °C, 10 min). Sections were incubated with primary antibodies at 4 °C overnight (Table 2). The labelled streptavidin–biotin peroxidase method (UltraTech HRP Streptavidin–Biotin Detection System, PN IM2391; Immunotech, Marseille, France) and the EnVision+ System HRP-labelled polymer (anti-mouse and anti-rabbit, DAKO, Carpinteria, CA, USA) were used. Positive reactions were detected as brown colouration with 3,3′-diaminobenzidine tetrahydrochloride (Dojindo, Kumamoto, Japan). Sections were then counterstained with haematoxylin.

**Table 2:** Primary antibodies used in this study.

Antibody (clone)	Species, isotype	Source	Dilution	Antigen retrieval
Podoplanin (D2-40)	Mouse, IgG1κ	DAKO, Carpinteria, CA, USA	× 50	pH 6.0, autoclave
LYVE1 (EPR21857)	Rabbit, IgG	Abcam, Cambridge, UK	× 2000	pH 9.0, autoclave
CD68 (PG-M1)	Mouse, IgG1κ	DAKO, Carpinteria, CA, USA	× 50	pH 6.0, autoclave
CD68 (KP-1)	Mouse, IgG3κ	DAKO, Carpinteria, CA, USA	× 50	pH 6.0, autoclave
CD163 (10D6)	Mouse, IgG1	Leica Biosystems, Eisfeld, Germany	× 50	pH 6.0, autoclave
CD206 (5C11)	Mouse, IgG1κ	Abnova, Taipei, Taiwan	× 200	pH 6.0, autoclave
HO-1 (D-8)	Mouse, IgG1	Santa Cruz, Dallas, TX, USA	× 50	pH 6.0, autoclave
TTF-1 (8G7G3/1)	Mouse, IgG	DAKO, Carpinteria, CA, USA	× 100	pH 9.0, autoclave
c-Maf	Rabbit, polyclonal	Abcam, Cambridge, UK	× 50	pH 8.0, autoclave
MMP-2	Rabbit, polyclonal	Abcam, Cambridge, UK	× 100	pH 6.0, autoclave

The following items (a, b) were examined: (a) The number of cross-sections of podoplanin (D2-40)^+^ and LYVE1^+^ lymphatic vessels was counted in three different areas: inside the tumour, on the border, and outside the tumour (for both PTC and FTC). A 0.5 mm width on both sides of the tumour border (total 1 mm width) was defined as the border area. The tumour part inside the border was regarded as inside the tumour, and the nontumourous part outside the border was regarded as outside the tumour. A luminal structure with an endothelial cell lining being immunopositive was defined as a lymphatic vessel. Podoplanin (D2-40) and LYVE1 respond to the lymphatic endothelium but not vascular endothelial cells. The number of cross-sections of the lymphatic vessel was counted manually in 5 fields of a 20 × microscopic power field. (b) The area of lymphatic endothelial cells expressing podoplanin (D2-40) or LYVE1 was calculated.

### Double IHC

Double IHC was also performed as previously described^23,24^. The conditions for sections, blocking, antigen retrieval and secondary antibodies were the same as those for single IHC. Positive reactions were detected as brown colouration with 3,3′-diaminobenzidine tetrahydrochloride, the Vulcan Fast Red Chromogen Kit 2 (BIOCARE MEDICAL, Concord, CA, USA) and the HistoGreen Substrate Kit for Peroxidase (Linaris, Heidelberg, Germany). Sections were then counterstained with haematoxylin.

The following three items (c, d) were examined: (c) The number of cells positive for each macrophage marker around the lymphatic vessels in the border area in PTC and FTC was counted in 5 fields obtained at a 40 × microscopic power. (d) The number of cells positive for M1 and M2 markers inside and outside the areas of lymphatic invasion in PTC was counted. In this study, CD68 (PG-M1 or KP-1)^+^CD163^-^CD206^-^ cells were defined as M1, and CD163^+^CD206^+^ cells were defined as M2. Specimens in which TTF-1^+^ PTC cells were observed in D2-40^+^ lymphatic vessels were designated as containing areas of lymphatic invasion. Furthermore, the intraluminal area of the lymphatic vessel with lymphatic cancer invasion was considered inside the lymphatic wall, and the area surrounding the lymphatic vessel with lymphatic cancer invasion, 100 μm in width, was considered outside the lymphatic wall.

### Triple IHC

Triple IHC was also performed as previously described^23,24^. A positive reaction of the first antibody (D2-40) was detected as brown colouration with DAB, and a positive reaction of the second antibody (pan-macrophage marker [an antibody cocktail solution for CD68 and CD163]) was detected as light red colouration with Vulcan Fast Red. The HistoGreen Substrate Kit for Peroxidase (Linaris, Heidelberg, Germany) was used for colour detection of the third antibody (c-Maf). Counterstaining was performed with haematoxylin. Triple IHC was used to examine the number of intralymphatic M2 (panmacrophage marker [an antibody cocktail solution for CD68 and CD163]^+^c-Maf^+^) and non-M2 (panmacrophage marker^+^c-Maf^-^) cells with lymphatic cancer invasion.

### Reverse transcriptase polymerase chain reaction (RT-PCR)

The conditions used for RT-PCR were also as previously described^23^. Formalin-fixed and paraffin-embedded tissues from PTC samples (n = 5) were used for mRNA detection by RT-PCR. mRNA was extracted with a WaxFree RNA Extraction Kit (TrimGen, Sparks, MD, USA) according to the manufacturer’s protocol. mRNA (< 5 μg) was used as a template for the synthesis of cDNA (20 μl) with a PrimeScript II 1st strand cDNA Synthesis Kit (Takara Bio, Shiga, JAPAN). Three microlitres of cDNA was PCR amplified utilizing EmeraldAmp PCR Master Mix (Takara) and specific primers. The primer sequences were 5′-TACAGGATCATTGGCTACACACC-3′ (sense) and 5′-GGTCACATCGCTCCAGACT-3′ (antisense) for human MMP-2 (90 bp) and 5′-GCACCGTCAAGGCTGAGAAC-3′ (sense) and 5′-TGGTGAAGACGCCAGTGGA-3′ (antisense) for human glyceraldehyde-3-phosphate dehydrogenase (GAPDH; 138 bp) as the internal control. cDNA was amplified as follows: 94 °C for 10 min; 40 cycles of 94 °C for 30 s, 60 °C for 45 s and 72 °C for 30 s; and 1 cycle of 72 °C for 6 min in a Veriti 96-Well Thermal Cycler (Applied Biosystems, Foster City, CA, USA). The final PCR products were placed at 4 °C until electrophoresis. We used primers for human MMP-2 designed by PrimerBank. The PCR products were electrophoresed in a 4% agarose gel and stained with ethidium bromide.

### In situ reverse transcriptase polymerase chain reaction (in situ RT-PCR)

We examined PTC tissues to determine the localization of MMP-2 mRNA. The conditions used for in situ RT-PCR were as described elsewhere^23,25^. Briefly, dewaxed and rehydrated 5 μm paraffin sections were fixed in 4% paraformaldehyde for 4 h at room temperature. After fixation, the slides were incubated in pepsin (8 mg/ml) for 75 min at 37 °C. After protein digestion, the slides were air-dried and incubated in DNase digestion solution (DNase I recombinant, RNase-free, Roche Diagnostics, Mannheim, Germany) at 37 °C overnight.

After DNase digestion, a reverse transcriptase reaction was performed in the solution provided in the PrimeScript II 1st strand cDNA Synthesis Kit (Takara) according to the manufacturer’s protocol. Coverslips were placed over the solutions in the DNase digestion and reverse transcriptase reactions to prevent evaporation. Slide sealers (Takara) were placed on the slides around the tissue prior to PCR.

The in situ RT-PCR mixture consisted of PCR buffer with 15 mM MgCl_2_, PCR digoxigenin (DIG) labelling mix, Taq DNA polymerase and primer pairs for MMP-2 (the same primer pairs used for RT-PCR). A total of 125 μl of PCR mix was applied to the tissue sections, which were then sealed with slide sealers. Slides were placed into a MasterCycler PCR thermal cycler (Eppendorf, Hamburg, Germany) for 1 cycle of 94 °C for 10 min; 30 cycles of 94 °C for 30 s, 60 °C for 45 s and 72 °C for 30 s; and 1 cycle of 72 °C for 6 min. The location of DIG incorporated into PCR amplicons was detected with an alkaline phosphatase assay using anti-DIG-alkaline phosphatase Fab fragments (1:100 dilution) (Roche Diagnostics), which were incubated with the samples for 30 min at 37 °C. The in situ PCR product was detected using Vulcan Fast Red and counterstained with haematoxylin. Tissue sections without reverse transcriptase reactions were used as negative controls.

### Statistical analysis

To calculate the cell count and area in immunohistochemical sections, we used quantification by digital image analysis (HALO imaging analysis software; Indica Labs, Corrales, NM). The Mann–Whitney U test was performed to analyse the difference between the two groups with Microsoft Excel 2013 (Microsoft, Redmond, WA, USA). Spearman's rank correlation coefficient was determined to examine the correlation between the two groups. JMP version 14 (SAS Institute, Tokyo, Japan) was used to plot the individual data. A *p* value less than 0.05 was considered significant in all tests.

## Supplementary Information

Supplementary Figure.

## Data Availability

The datasets generated and/or analysed during the current study are available from the corresponding author upon reasonable request.
