# Supplementary material for: Potential role of M2 TAMs around lymphatic vessels during lymphatic invasion in papillary thyroid carcinoma
Source: Sci Rep. 2021 Jan 13;11:1150. doi: 10.1038/s41598-020-80694-3 (PMC7806843; doi:10.1038/s41598-020-80694-3)
Supplement: Supplementary file 1 — Supplementary Figure. [file 41598_2020_80694_MOESM1_ESM.docx]

**Supplementary information**

Supplementary Figure 1

Potential role of M2 TAMs around lymphatic vessels during lymphatic invasion in papillary thyroid carcinoma

Takanobu Kabasawa, Rintaro Ohe, Naing Ye Aung, Yuka Urano, Takumi Kitaoka,

Nobuyuki Tamazawa, Aya Utsunomiya, and Mitsunori Yamakawa


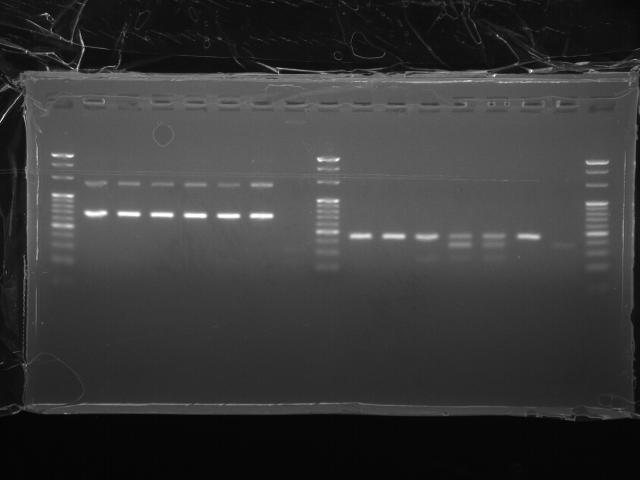


Supplementary Figure 1; Full-sized gel image of Fig. 6a
